# The causal relationship between gut microbiota and type 2 diabetes: a two-sample Mendelian randomized study

**DOI:** 10.3389/fpubh.2023.1255059

**Published:** 2023-09-22

**Authors:** Kewang Sun, Yan Gao, Huaqing Wu, Xiangyan Huang

**Affiliations:** ^1^School of Medical Laboratory, Weifang Medical College, Weifang, China; ^2^Department of Blood Transfusion, The 960th Hospital of the PLA Jonit Logistics Support Force, Jinan, China; ^3^Department of General Medicine, The 960th Hospital of the PLA Jonit Logistics Support Force, Jinan, China; ^4^School of Mathematics and Statistics, Beijing Technology and Business University, Beijing, China

**Keywords:** Mendelian randomized study, gut microbiota, type 2 diabetes, causal inference, genetic variation

## Abstract

**Background:**

Type 2 diabetes mellitus (T2DM) is a commonly observed metabolic anomaly globally, and as of the present time, there's no recognized solution. There is an increasing body of evidence from numerous observational studies indicating a significant correlation between gut flora and metabolic disease progression, particularly in relation to T2DM. Despite this, the direct impact of gut microbiota on T2DM isn't fully understood yet.

**Methods:**

The summary statistical figures for intestinal microbiota were sourced from the MiBioGen consortium, while the summary statistical data for T2DM were gathered from the Genome-Wide Association Studies (GWAS) database. These datasets were used to execute a two-sample Mendelian randomization (MR) investigation. The Inverse Variance Weighted (IVW), Maximum Likelihood, MR-Egger, Weighted Median, and Weighted Models strategies were employed to assess the impact of gut microbiota on T2DM. Findings were primarily obtained using the IVW technique. Techniques like MR-Egger were employed to identify the occurrence of horizontal pleiotropy among instrumental variables. Meanwhile, Cochran's Q statistical measures were utilized to assess the variability or heterogeneity within these instrumental variables.

**Results:**

The outcomes from the IVW analysis demonstrated that the genus *Alistipes* (OR = 0.998, 95% confidence interval: 0.996–1.000, and *P* = 0.038), genus *Allisonella* (OR = 0.998, 95% confidence interval: 0.997-0.999, *P* = 0.033), genus *Flavonifractor* (OR = 0.995, 95% confidence interval: 0.993–0.998, *P* = 3.78 × 10^−3^), and genus *Haemophilus* (OR = 0.995, 95% confidence interval: 0.993–0.998, *P* = 8.08 × 10^−3^) all acted as defense elements against type 2 diabetes. Family *Clostridiaceae1* (OR = 1.003, 95% confidence interval: 1.001–1.005, *P* = 0.012), family *Coriobacteriaceae* (OR = 1.0025, 95% confidence interval: 1.000–1.005, *P* = 0.043), genus *Actinomyces* (OR = 1.003,95% confidence interval: 1.001–1.005, *P* = 4.38 × 10^−3^), genus *Candidatus Soleaferrea* (OR = 1.001,95% confidence interval: 1.000–1.002 *P* = 0.012) were risk factors for type 2 diabetes. False Discovery Rate correction was performed with finding that genus.*Allisonella*, genus*.Alistipes*, family *Coriobacteriaceae*and T2DM no longer displayed a significant causal association. In addition, no significant heterogeneity or horizontal pleiotropy was found for instrumental variable.

**Conclusion:**

This MR study relies on genetic variation tools to confirm the causal effect of genus *Flavonifractor*, genus *Haemophilus*, family *Clostridiaceae1*, genus *Actinomyces* and genus *Candidatus Soleaferrea* on T2DM in the gut microbiome, providing new directions and strategies for the treatment and early screening of T2DM, which carries significant clinical relevance. To develop new biomarkers and better understand targeted prevention strategies for T2DM, further comprehensive investigations are required into the protective and detrimental mechanisms exerted by these five genera against T2DM.

## 1. Introduction

As society progresses and living conditions improve, increased obesity rates, declining air quality, and an aging population are risk factors for a range of chronic metabolic diseases. One of such a globally prevalent disease is diabetes mellitus (DM), which includes type 2 diabetes mellitus (T2DM) (1). According to several studies, the global prevalence of diabetes surpassed 460 million adults in 2019. Furthermore, considering the current growth rate, projections suggest that the number of individuals living with diabetes will double by the year 2045 (2). As the prevalence of type 2 diabetes continues to increase over time, complications such as systemic macrovascular and microangiopathy can occur if left untreated (3). This can place enormous economic pressure on global health systems. Therefore, it is essential to explore the pathogenesis of type 2 diabetes and the search for more effective treatments.

Gut flora, the collective term for the vast microbial ecosystem residing in our intestinal tract, boasts an impressive count of roughly 40 trillion bacteria and a gene count that exceeds the human's by approximately 150-fold (4). Typically, the intestinal flora, also known as the gut microbiota, maintains a dynamic and harmonious relationship with both the host body and the external surroundings. This intricate equilibrium remains constant over time, reflecting the intricate interplay between the gut microbiota, the human host, and the surrounding environment. Nevertheless, any disruption to this balance could lead to compromised host functions. It's reported that nearly 95% of all health conditions have some link to gut flora (5). This connection extends beyond gastrointestinal and metabolic disorders, as the gut flora is also implicated in various systemic ailments, including neurological, respiratory, cardiovascular, and cancerous diseases (6–11). The communication between the gut microbiota and the host primarily occurs through various small molecular metabolites. These metabolites, including but not limited to short-chain fatty acids, bile acids, tryptophan, and amino acids, play a significant role in influencing the progression of diseases. They serve as crucial messengers in conveying signals and exerting effects on the overall health and wellbeing of the host organism (12).

Recent studies have shown that in addition to poor dietary habits and impaired islet function, intestinal flora disorders may also contribute to T2DM (13), and that there are some probiotics in the intestinal flora that can effectively control blood glucose in patients with T2DM (14). Controlling for confounding factors like age, gender, and lifestyle in previous observational studies has been challenging, leading to less reliable and accurate experimental outcomes.

The Mendelian Randomization (MR) employed in this study serves as a tool for epidemiological scrutiny, which gauges the link between genetic variants and outcomes such as disease manifestation or mortality, based on genetic alterations tied to exposure elements. Essentially, it utilizes genetic information as a conduit to investigate the causal relationship between exposure and outcomes. MR is a potent instrument for making causal deductions, effectively circumventing the confounding bias often seen in traditional epidemiological research (15). In the context of this study, we employed Single Nucleotide Polymorphisms (SNPs) as the instrumental variables. By employing these SNPs, we were able to establish a comprehensive mapping of the intricate relationship, shedding light on the potential mechanisms underlying the development of type 2 diabetes in relation to the characteristics of the gut microbiota.

## 2. Methods

### 2.1. Data sources

In our research, we conducted two-sample MR analyses, designating gut microbiota as the exposure and type 2 diabetes as the outcome variable. The exposure data was sourced from MiBioGen (https://mibiogen.gcc.rug.nl/), a consortium that aggregates 16s ribosomal RNA (rRNA) gene sequencing and genotyping information from 18,340 participants across 24 countries, including nations like the US and Canada. This was performed on 131 gut microbiota genera exhibiting mean abundances exceeding 1%, as per recent observational investigations (12). According to a recent article summarized the intestinal flora that may be associated with type 2 diabetes (13–15), we opted for 6 families and 14 genera that are potentially linked with type 2 diabetes to serve as an exposure factor in this MR study (refer to additional documents 1 for more specifics). Our results stem from consolidated data sourced from the IEU Open GWAS database (https://gwas.mrcieu.ac.uk/datasets/ukb-b-13806/). The data includes a European population with a comprehensive sample size of 462,933, encompassing 2,972 individuals diagnosed with type 2 diabetes and 459,961 control subjects. The total number of SNPs was 9,851,867. The patients participating in the database were ethically approved. By utilizing freely available data for our research and publication, we successfully circumvented potential ethical concerns and other potential conflicts of interest. Our study leverages open-source data, thereby ensuring its freedom from any ethical dilemmas or competing interests.

### 2.2. Experimental design

In an effort to explore the cause-and-effect link between intestinal microflora and T2DM, a bi-sample Mendelian Randomization study was implemented, leveraging data from the MiBioGen consortium and the compiled dataset from GWAS. The instrumental variables (IVs) were initially subjected to a screening process. For an IV to be utilized in MR, it had to meet three primary assumptions: (1) Correlation: the SNPs were robustly linked to exposure; (2) Exclusivity: the SNPs did not correlate with the outcome; (3) Independence: the SNPs showed no association with confounding factors (16–18). A pooling process was conducted for SNP loci with *P* < 1 × 10^−5^, establishing a linkage disequilibrium (LD) with r^2^ <0.05 and a genetic distance of 10 MB, then SNPs with small *P*-values were chosen after echo sequence SNPs were eliminated. This process ensured that IVs fulfilled assumption (1). The potency of the IVs was gauged by calculating the F statistic, using the formula:


$$F\;=\;\frac{\beta_{exposure}^{2}}{SE_{exposure}^{2}}$$

where in β denotes the effect size of the SNP on exposure and SE represents β's standard error.

**Figure 1:**
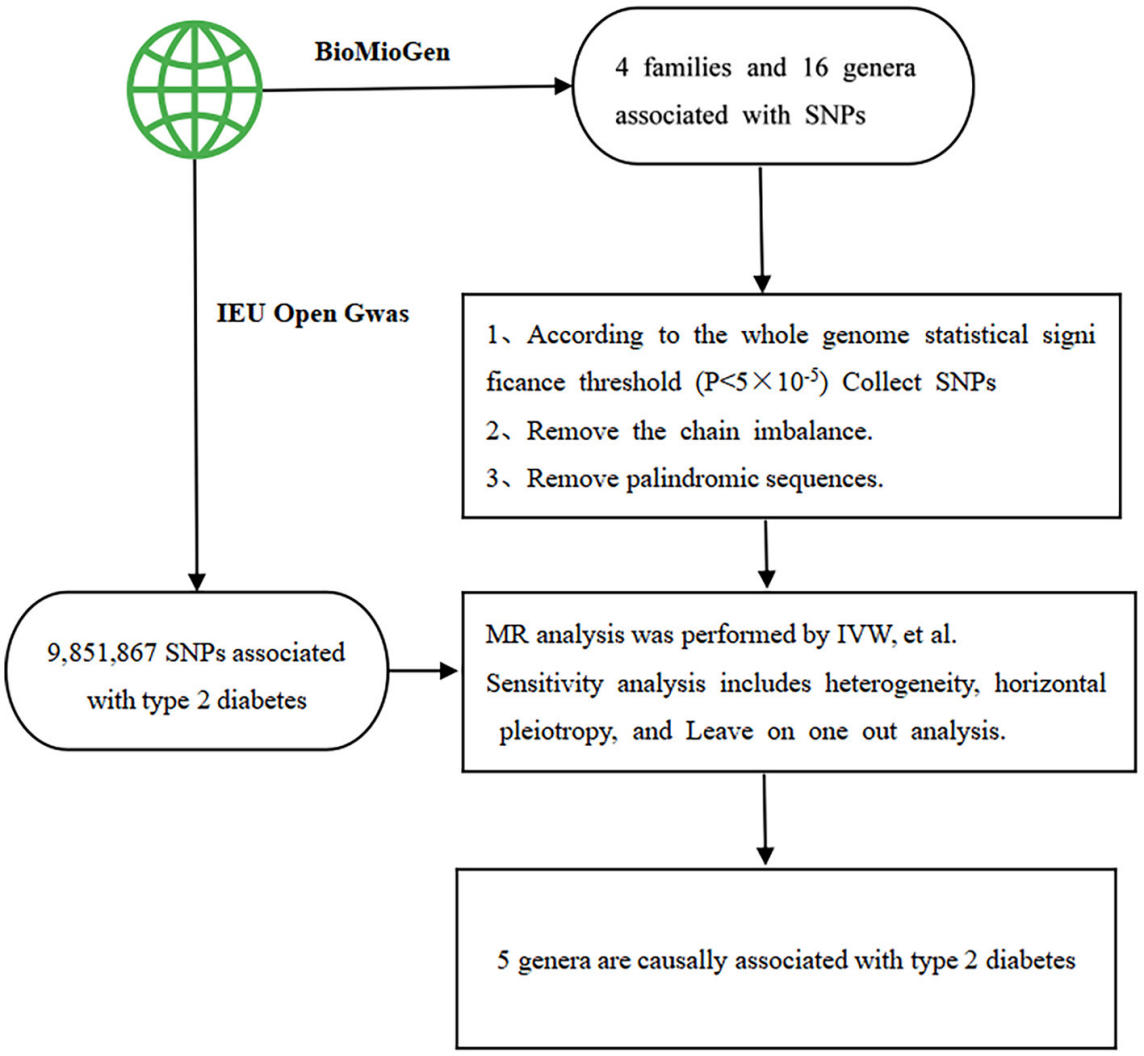
The flow chart outlines the study design and the process of Mendelian randomization (MR) analysis. SNP stands for single nucleotide polymorphism, which serves as the instrumental variables. IVW represents inverse-variance weighted, a method employed in the analysis.

An F statistic >10 was considered indicative of insignificant weak instrumental bias. Subsequently, data were extracted from both databases and compiled such that the impact values for exposure and outcome corresponded to the same effect allele. The identified SNPs linked with each genus underwent analysis via various statistical methods to deduce causal associations between gut flora and T2DM across the 6 families and 14 genera. Finally, to fulfill MR assumptions (2) and (3), SNPs directly linked to confounding factors and outcomes were excluded using the phenoscanner website (http://www.phenoscanner.medschl.cam.ac.uk/). Figure 1 offers a flow diagram outlining the study design and the MR analysis procedure.

### 2.3. Statistical analysis

Within the framework of this research, a diverse array of methodologies was employed to explore the potential causal relationship between the gut microbiota and T2DM. These techniques encompassed an assortment of approaches, including but not limited to inverse variance weighted (IVW), Simple mode, MR-Egger regression, weighted median (WM), and weighted model (WME). The utilization of these multiple approaches allowed for a comprehensive investigation into the prospective causal linkages between the intestinal microflora and the occurrence of T2DM. Primarily, the IVW method was utilized as a leading causal effect estimator in MR research, demonstrating robust causal relationship detection and high testing efficacy (19). However, the specific requirement of the IVW method is that genetic variation influences the target outcome solely via exposure under study. In the context of the MR-Egger method, an intercept term is considered, and its presence is utilized to assess pleiotropy. If the intercept term approaches zero, the MR-Egger regression model aligns closely with IVW. But, if the intercept term substantially diverges from zero, it indicates the possible existence of horizontal pleiotropy among these IVs (19). The weighted median method (WME) serves as a complement to MR-Egger, offering unbiased estimations even when up to 50% of the weights derive from invalid instrumental variables (20). Given that multiple tests enhance the probability of type I errors, a false discovery rate (FDR) correction was applied.

### 2.4. Sensitivity analysis

To confirm the dependability of our outcomes, we performed sensitivity checks to gauge the sturdiness of the results, possible biases (like genetic pleiotropy and data diversity), and the impact of particular instrumental variables on the result variable (21). The MR-Egger approach was utilized to evaluate the existence of multiple-testing issues within the data, while the Cochran Q test was employed to inspect for disparities among IVs, where larger discrepancies signaled increased heterogeneity. The leave-one-out technique was implemented to determine the impact of a singular SNP on the outcome. This was accomplished by sequentially excluding SNPs and then computing the combined effect of the remaining SNPs, thereby gauging the extent of influence and stability. In the context of this research, various techniques were employed to explore the potential causal relationship between the characteristics of the gut microbiota and the development of type 2 diabetes. These methodologies included the utilization of inverse variance weighted (IVW), Simple mode(SM), MR-Egger regression, weighted median (WM), and weighted model (WME) approaches. By leveraging this diverse set of methodologies, we were able to comprehensively investigate the prospective causal association between the intestinal microflora and the emergence of type 2 diabetes. To present the results, the outcomes were expressed as odds ratios (OR) accompanied by their corresponding 95% confidence intervals (95% CI). Statistical significance was determined by a *p* < 0.05. All statistical computations for this study were performed using version 4.3.0 of the R software, developed by the R Foundation for Statistical Computing in Vienna, Austria. Specifically, the MR study was conducted using the TwoSampleMR package (version 0.5.6), which proved to be a valuable tool in our analyses.

## 3. Results

### 3.1. Strength of genetic instruments

In our efforts to discern the causal influence of gut microbiota on T2DM, we amalgamated SNPs following a genome-wide significance criterion (*P* < 1 × 10^−5^), established a linkage disequilibrium cutoff at 0.05, and set a consolidation window at 10 Mb. We also eliminated palindromic sequences. Each instrumental variable (IV) boasted an F-statistic exceeding 10, signifying the absence of weak instrumental bias. Further details can be found in additional documents 2.

### 3.2. Association of intestinal flora with T2DM

**Figure 2:**
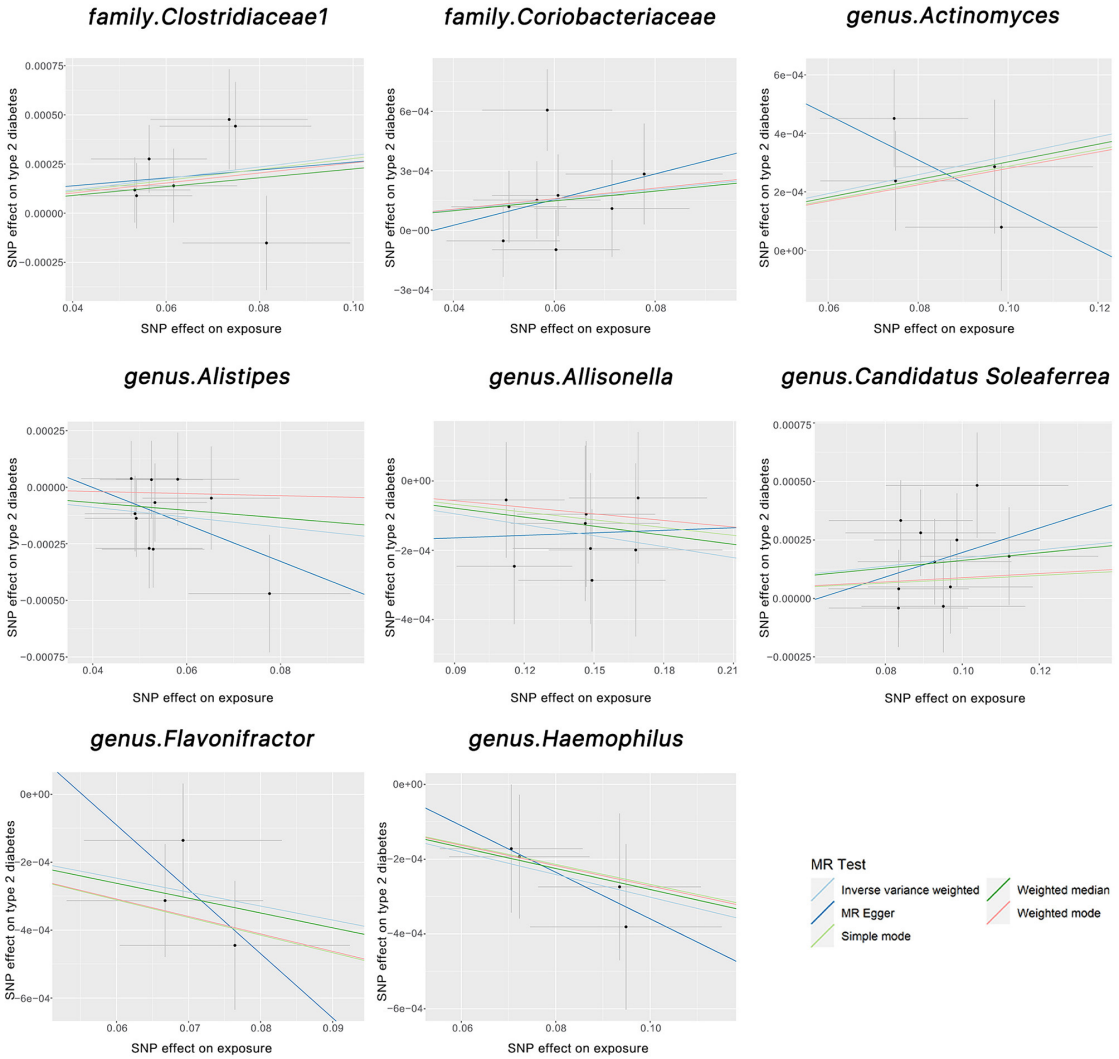
The scatter plots above illustrate the causal association between gut microbiota and T2DM. The light blue, light green, dark blue, green, and pink lines correspond to the Inverse Variance Weighted, Simple Mode, MR-Egger, Weighted Median, and Weighted Model methods, respectively.

**Figure 3:**
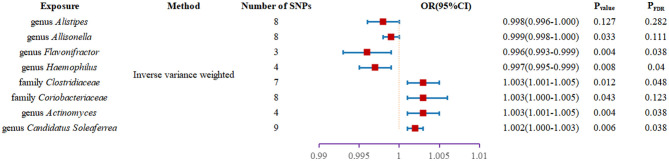
Forest plot of the causal association between gut microbiota and T2DM.

We found two families and six genera to be causally associated with T2DM using MR methods, as shown in Figures 2, 3, Supplementary Table 1. After FDR correction and exclusion of SNPs directly associated with body fat percentage, body weight, and T2DM (rs1689282, rs8130320, rs6494306), IVW results showed genus *Flavonifracto*r (OR = 0.995, 95% CI: 0.993–0.998, *P*_FDR_ =0.040), genus.*Haemophilus* (OR = 0.995, 95% CI: 0.993–0.998, *P*_FDR_ =0.038), family.*Clostridiaceae1* (OR = 1.003, 95% CI: 1.001–1.005, *P*_FDR_ = 0.048), genus.*Actinomyces* (OR = 1.003, 95% CI: 1.001–1.005, *P*_FDR_ = 0.038), genus.*Candidatus Soleaferrea* (OR = 1.001, 95% CI: 1.000–1.002, *P*_FDR_ = 0.038) were still causally associated with T2DM. In contrast, genus.*Allisonella* (OR = 0.998, 95% CI: 0.997–0.999, *P*_FDR_ = 0.111), genus.*Alistipes* (OR = 0.998,95% CI: 0.996–1.000, *P*_FDR_=0.127), and family. *Coriobacteriaceae* (OR = 1.0025, 95% CI: 1.000–1.005, *P*_FDR_ = 0.124) were no longer causally significantly associated with T2DM.

### 3.3. Sensitivity analyses

**Figure 4:**
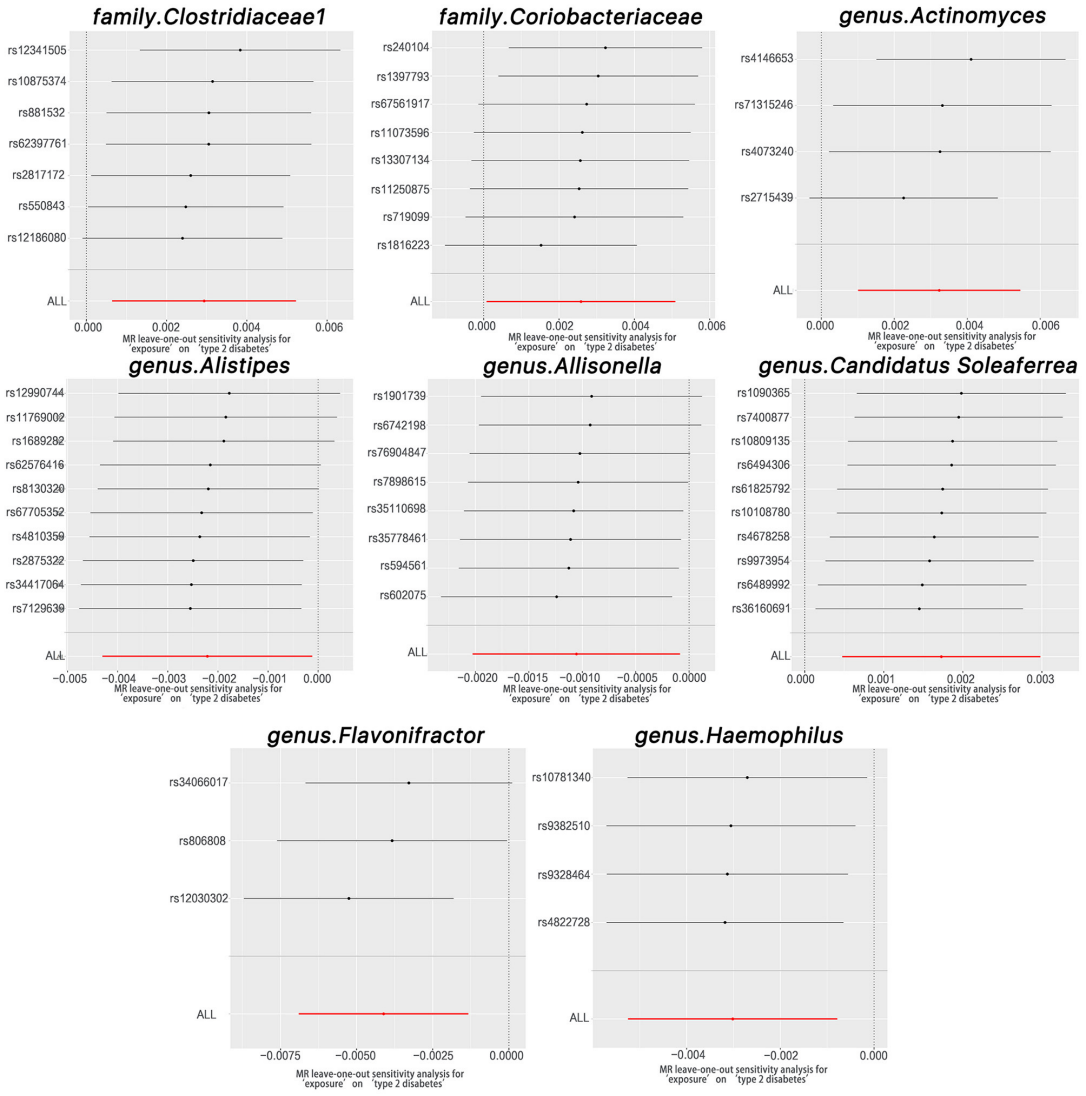
Leave-one-out plots for the causal association between gut microbiota and T2DM.

The Cochran Q test indicated no heterogeneity within the instrumental variables, as the *P*-values for both the IVW and MR Egger analyses surpassed 0.05. Moreover, the multi-allelic assessment revealed no heterogeneity in the instrumental variables as the *P*-value for all seven genera exceeded 0.05 (Table 1). The leave-one-out exploration did not identify any SNPs significantly influencing the correlation between the gut microbiome and T2DM, as depicted in Figure 4.

**Table 1:** MR estimates for the association between gut microbiota and T2DM.

**Exposure**	**Outcome**	**Number of SNPs**	**MR-Egger regression**		**Heterogeneity analyses**		
			**Intercept**	**P_intercept**	**Method**	**Q**	**Q_pval**
genus*.Allisonella*	Diabetes mellitus type 2	8	−1.86 × 10^−4^	0.71	MR-Egger	1.53	0.96
					IVW	1.69	0.97
genus.*Flavonifractor*		3	1.04 × 10^−3^	0.66	MR-Egger	0.98	0.32
					IVW	1.32	0.52
genus.*Haemophilus*		4	2.63 × 10^−4^	0.73	MR-Egger	0.11	0.95
					IVW	0.26	0.97
family.*Clostridiaceae*		7	5.86 × 10^−5^	0.91	MR-Egger	5.36	0.37
					IVW	5.37	0.50
family.*Coriobacteriaceae*		8	−2.34 × 10^−4^	0.70	MR-Egger	7.69	0.28
					IVW	7.49	0.36
genus.*Actinomyces*		4	9.24 × 10^−4^	0.32	MR-Egger	1.16	0.55
					IVW	2.80	0.42
genus.*Candidatus Soleaferrea*		10	−3.27 × 10^−4^	0.62	MR-Egger	6.36	0.61
					IVW	6.62	0.68

## 4. Discussion

In this study, we executed a bi-sample Mendelian randomization (MR) investigation, using data from the MiBioGen consortium and the consolidated GWAS dataset, to appraise the cause-and-effect relationship between particular intestinal microflora and T2DM. We identified two genera as protective factors for T2DM, namely genus.*Flavonifractor* and genus.*Haemophilus*; and three genera as risk factors for T2DM, namely family.*Clostridiaceae*, genus.*Actinomyces*, and genus. *Candidatus Soleaferrea*.

Numerous recent studies have consistently reported a strong correlation between gut microbiota and type T2DM (13, 22–24). Specifically, one study has identified ~ 60,000 molecular markers associated with diabetes through conducting a genomic association analysis of 345 genera in the gut flora. This underlines the disparities in gut microbial makeup at the molecular level between individuals with diabetes and those without the disease (23). In this study, we identified genus.*Flavonifractor* as a protective factor for T2DM. genus.*Flavonifractor* is an important species affecting gut health and its abundance is negatively correlated with obesity (25). Another related study reported that genus.*Flavonifractor* is a butyrate producer (26). Butyrate is a type of Short-Chain Fatty Acid (SCFA), which is an organic carboxylic compound containing 1-6 carbon atoms. The human body is primarily abundant with acetic acid (C2), propionic acid (C3), and butyric acid (C4) as part of its SCFA composition (27). These SCFAs have a regulatory role in various systems such as gastrointestinal, neurological, endocrine, and hematological. Numerous studies suggest that SCFAs have a crucial role in preserving intestinal health and improving the outcomes of many non-communicable illnesses, including cancer (28). It has been found that SCFAs stimulate the release of glucagon-like peptide 1 (GLP-1) and peptide tyrosine-tyrosine (PYY) in rat and mouse models (29). GLP-1 encourages insulin production, improves insulin sensitivity, inhibits gastric emptying, and reduces gut motility; PYY modulates gut motility, slows gastric emptying, enhances satiety, and lessens food consumption (30). SCFAs can also ameliorate insulin resistance by fostering the synthesis and release of PYY and GLP-1 in the intestinal epithelial gland cells (31, 32). Studies on mice demonstrated that butyric acid treatment lowers fasting blood glucose and insulin levels while improving insulin sensitivity (32).

For the other four genera identified in this study besides genus.*Flavonifractor*, relevant studies also support the results of this study. One research study noted a reduced presence of the genus *Haemophilus* in the intestines of patients diagnosed with T2DM (33). Significantly, genus *Haemophilus*, a bacterium harmful to humans, can lead to primary septic infections. Recent research has found its existence is linked to oral, head and neck, pancreatic, and stomach cancers (34–36). In several clinical and animal studies, T2DM has related to chronic inflammation (37–39). The genus *Candidatus Soleaferrea* is linked with intestinal inflammation and persistent low-level inflammation. The initiation of the immune system, leading to an inflammatory response, is linked with the development of T2DM, thereby establishing that genus Soleaferrea is a T2DM risk factor. In mouse models, Clostridiaceae1 may be critical to the abnormal metabolism observed in type 2 diabetes (40). Genus *Coriobacteriaceae* and genus *Actinomyces* were relatively elevated in abundance in T2DM patients. In summary, the results from previous studies are consistent with the conclusions reached in this study.

It is well known that T2DM cannot be completely cured under the current medical conditions, so it is crucial to prevent the occurrence of T2DM. Several reports have shown that intestinal dysbiosis and a decrease in short-chain fatty acid-producing bacteria increase the risk of type 2 diabetes mellitus (13, 41). According to the results of this MR study, we can implement secondary prevention for people exposed to T2DM risk factors (42). Specifically, we can carry out targeted screening of intestinal flora in people exposed to T2DM risk factors, such as the elderly or obese people, in order to detect diseases early and establish treatment mechanisms. Through screening, we urge them to maintain good dietary habits, regularly use appropriate probiotic supplements to maintain the balance of Gut microbiota, and maintain normal Short-chain fatty acid metabolism to effectively prevent type 2 diabetes.

Traditional observational studies measure environmental exposure factors that are associated with behavioral, social, and psychological factors, resulting in bias. MR, however, is not affected by these confounding factors. Relative to other methods, MR has less measurement error in relation to its effects, and data from the GWAS are relatively easy to obtain and less costly when conducting MR analyses.

## 5. Limitations

Initially, it's important to consider that allele frequency and disease prevalence can differ across various populations, hence, population stratification could introduce a confounding element in Mendelian random analysis, especially if the study population is diverse (43). Secondly, the information on SNPs in T2DM patients sourced in this study was derived from a European populace, hence it may not be universally applicable to all ethnicities. Detailed demographics such as age and gender were also not provided. Lastly, it's essential to note that MR is capable of identifying genera having a causal link between the presence of certain gut flora and T2DM, however, it doesn't delve deeper into the specific biomolecular mechanisms.

## 6. Conclusions

To summarize, this two-sample MR study's findings offer genetic proof that the existence of genus *Flavonifractor*, genus *Haemophilus*, family *Clostridiaceae1*, genus *Actinomyces*, and genus *Candidatus Soleaferrea* in our intestines is causatively linked to T2DM's onset. This is clinically significant as it provides fresh avenues and novel strategies for T2DM's treatment and early detection. Nonetheless, additional research is required to clarify the specific protective and detrimental mechanisms of these five genera against T2DM. Moreover, considering this study was only conducted on a European population, future studies should incorporate multi-ethnic, multi-age, and gender-specific MR research to arrive at comprehensive conclusions.

## Data availability statement

The data presented in this study is deposited in publicly available datasets. This data can be found at: gut bacteria from MiBioGen (data available at: https://mibiogen.gcc.rug.nl/), and Type 2 diabetes from MRC Integrative Epidemiology Unit (https://gwas.mrcieu.ac.uk/datasets/ukb-b-13806/).

## Author contributions

KS: Data curation, Software, Writing—original draft, Writing—review and editing. YG: Formal Analysis, Methodology, Supervision, Writing—original draft, Writing—review and editing. HW: Data curation, Software, Validation, Writing—review and editing. XH: Conceptualization, Methodology, Validation, Writing—original draft, Writing—review and editing.
